# Cellulitis in aged persons: a neglected infection in the literature

**DOI:** 10.11604/pamj.2017.27.160.12007

**Published:** 2017-06-30

**Authors:** Anis Mzabi, Wafa Marrakchi, Zeineb Alaya, Fatma Ben Fredj, Amel Rezgui, Elyès Bouajina, Chedia Laouani Kechrid

**Affiliations:** 1 Department of Internal Medicine, Sahloul Hospital, Faculty of Medicine of Sousse, Sousse, Tunisia; 2 Department of Rheumatology, Farhat Hached Hospital, Faculty of Medicine of Sousse, Sousse, Tunisia

**Keywords:** Cellulitis, aged person, diabetes, necrotizing fasciitis, antibiotic, surgery

## Abstract

Cellulitis is a frequent soft tissue and skin infection. The lower limbs are affected in 70 to 80% of cases. Cellulitis in aged persons is not yet well described in literature. A retrospective descriptive study conducted in the Internal Medicine Department of Sahloul hospital in Sousse in Tunisia. It included patients whose age was up to 65 years old admitted into hospital for cellulitis of the legs, the arms or the face. One hundred fifty eight patients with a mean age of 73 years old (range: 65 to 94 years old) were included. Female to male sex ratio was 0.68. Among them, we noted diabetes mellitus in 81 cases (50.6%). The infection was located in the lower limbs in 155 cases (98%), in the face in two cases (1.3%) and in the upper limb in one case (0.7%). Twenty one patients (13.3%) presented with severe cellulitis and one presented with necrotizing fasciitis. All patients received intra venous antibiotic therapy. Surgical treatment was indicated in 14 cases. Cefazolin was prescribed in 77 cases (48%). Favorable evolution was noted in 144 patients (91.1%). Forty four patients (27.8%) received prophylactic antibiotics. Prevention of skin and soft tissue infection is a crucial step to preserve health in aged persons.

## Introduction

Cellulitis is a skin and soft tissue infection mostly caused by gram stain positive cocci especially streptococcus pyogenes and staphylococcus aureus [1–5]. Currently, the distribution of cellulitis has changed: the lower limbs are affected in 70 to 80% of cases, and the face is affected in 5 to 20% of cases [2–4]. Aged persons are frequently predisposed to this infection [2]. Cellulitis in this age range is associated with significant morbidity and health care costs [4]. Their characteristics in aged persons are not yet well determined. This infection in this age range is not well described in literature. The aims of our study are: to analyse the clinical forms of cellulitis in aged persons; To determine the microbial features of this infection; To evaluate the antibiotic therapy; To plan for optimal management.

## Methods

This retrospective study was conducted at the Internal Medicine and Rheumatology Department in Sahloul Hospital in Sousse in Tunisia over a period of 18 years (1999-2016). It was based on the medical records of patients hospitalized for cellulitis. The study population was made up of all the patients whose age was superior to 65 years old, admitted into hospital for cellulitis of the legs, the arms or the face. The parameters of interest in the study were: epidemiological, clinical, microbial and therapeutic aspects. Data were recorded and analyzed statistically using the software SPSS evaluating free version.

## Results

### Epidemiological characteristics of cellulitis

One hundred fifty eight patients comprising 94 men (59.5 %) and 64 women (40.5 %) (M/F sex ratio: 0.68) with a mean age of 73 years old (range: 65 to 94 years old) were enrolled. Among them, we noted diabetes mellitus in 81 cases (50.6%), smoking in 51 cases (32.3%), obesity in 52 cases (33%), heart failure in 25 cases (15.8%), recurrent cellulitis in 14 cases (8.8%) and alcoholism in two cases (1.2%). Thirteen patients (8.2%) received corticosteroids and four patients (2.5%) received anti-inflammatory treatment.

### Clinical presentation

**Figure 1:**
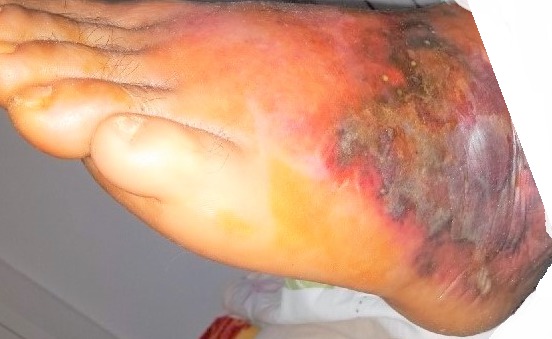
Necrotizing fasciitis

**Figure 2:**
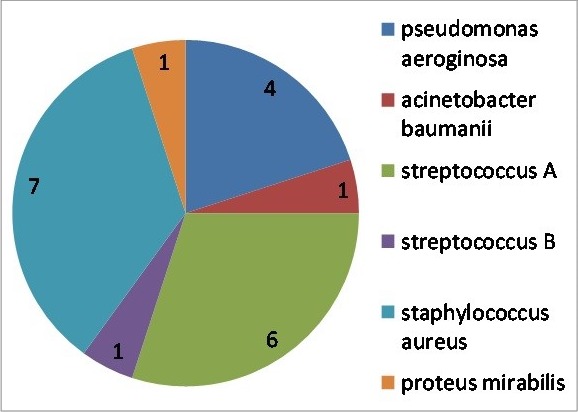
Microbial aspects of cellulitis

All patients had local signs of inflammation: erythema associated with pain and swelling. The infection was located in the lower limbs in 155 cases (98%), in the face in two cases (1.3%) and in the upper limb in one case (0.7%). Fever was noted in 59 cases (37%) with a mean temperature 38.9° (range: 38°-40.3°). Satellite lymph node was found in ten patients (6.3%). Twenty one patients (13.3%) presented with severe cellulitis and one presented with necrotizing fasciitis (Figure 1). Inflammatory syndrome was found in 143 cases (90%) the mean values of white blood cells, sedimentation rate and C-reactive protein were respectively 13 220 elements/mm^3^ 92 mm and 140 mg/l. Blood culture was done in 28 patients and among them only one blood culture was positive and it isolated streptococcus B. Bacteriological superficial samples from the primary lesion were positive in 23 cases (14.5%) (Figure 2). Risk factors were toe web intertrigo in 53 cases (46%), a wound in 28 cases (25%) and vascular disease in 11 cases (10%) (Table 1).

**Table 1:** Risk factors for cellulitis

Risk factor	Number of patients	% of patients
Intertigo	53	46
Wound	28	25
Cutaneous Ulcer	11	10
Arterial insuffiency	10	9,1
Lymphedema	7	6,4
Veinous insuffiency	7	6,4
Phlebitis	3	3

**Evolution** Favorable evolution was noted in 144 patients (91.1%). Complicated cases were: diabetic ketoacidosis in three patients (1.8%), necrotizing fasciitis in eight cases (5%), skin abscess and phlebitis in six cases (3.8%) each one. Recurrent cellulitis was retained in 52 cases (33%). It was the second episode in 31 cases (20%), the third episode in 12 cases (7.6%) and more than three episodes in eight cases (5.4%).

### Cellulitis treatment

### Antibitic therapy

All patients received intra veinous antibiotic therapy. Different protocols were: cefazolin in 77 cases (48%), Penicillin G associated with Penicillin M in 45 cases (28%), amoxicillin in 20 cases (12.6%), amoxicillin-clavulanic acid associated with fluorquinolone in 11 cases (7%) and Penicillin G in five cases (3%). Antibiotic oral relay was indicated in 52 cases: pristinamycin (31 cases), amoxicillin (10 cases), amoxicillin-clavulanic acid (7 cases) and erythromycin (4 cases). Forty four patients (27.8%) received prophylactic antibiotics. Among them, 43 patients (97.7%) received Benzathine Benzylpenicillin and one patient received erythromycin. The treatment of intertrigo was recommended in all patients having this pathology. Surgical treatment was indicated in 14 cases (8.8%): seven patients (50%) underwent excision of necrotic tissue, four patients (28.5%) underwent flattening of abscess, two patients (14.2%) underwent amputation and one patient (7.1%) underwent coetaneous graft.

## Discussion

Our analysis had been particularly interested with aged persons. It is explicated by the fact that this age range in our population is currently important. Few published studies had evaluated this infection in aged persons [1]. The soft tissue and skin infections are also frequent [1, 2]. They are a major problem of health care. Uncomplicated cellulitis are common infections and a frequent cause of hospital admission with considerable morbidity , costs and absenteeism [2, 6, 7] .The cellulitis of the lower leg is the most frequent [1]. It tends to recur in a substantial proportion of patients following an initial episode. Risk factors are well identified. The most important are tinea pedis or other toe web maceration or skin breaks, veinous oedema or lymphoedema, obesity and diabetes [2]. The other localisations are not frequent but are in the most cases more serious and hospitalization was recommended [1]. Aged persons are indeed concerned with cellulitis. They are fragile and their co-morbidities led to get the situation worse. They have multiple coexisting diseases such as diabetes, cardiac failure, vascular diseases and severe obesity which are risk factors for mortality from cellulitis [1]. In our study, the majority of patients had risk factors for severe cellulitis: diabetes in 50% of cases and obesity in 32.3%. Clinical presentation was typical with oedema, erythema and swelling [1]. However, all patients in our study had all criteria for hospitalization. They needed intra venous antibiotics in all cases. The failure of oral antibiotics is due to malabsoption described in aged patients. It has been suggested that aged patients have more severe cellulitis [4]. The risk of necrotizing fasciitis is estimated to be 13.3% according to our analysis. Thus, all clinical presentation should benefit from close monitoring in aged persons.

In our study, we had cases of decompensation of chronic diseases and local vascular complications like phlebitis. These results suggest that the decrease in activity due to the infection and the intra venous treatment could cause local vascular complications. Therefore, these data suggest that a simple cellulitis could increase the duration of hospitalization and the costs. The antibiotics were necessary in the management of cellulitis in aged persons [7]. Penicillin G and M, first generation cephalosporin had well results in this age range [7, 8]. However, we should pay attention to kidney, heart and drug-drug interactions. Polymedication, renal problems and heart failure may lead to renal failure. Sodium overload caused by penicillin G could generate heart failure or renal failure [9]. Aged patients take frequently anti-vitamin K and allopurinol. Drug drug interaction between antibiotics and these treatments could be a cause of death. Recurrent cellulitis was noted in 33% of cases [3, 5, 8]. The age generates venous insufficiency, lymphoedema and fungal local infections [5, 6]. These factors increase the risk of recurrence [8–10]. We should consider prophylactic treatment in all patients to prevent recurrence, hospitalization and to decrease morbid-mortality and costs [9]. However, in most cases, having an intramuscular treatment every two weeks could be associated with poor compliance. That is why sequential prophylactic treatment is recommended in aged patients [10]. The strong points of our study were that it included only aged patients. This infection in this age range is not well described in literature. It insists on complications, recurrence and costs. This should encourage authority to promote home care and to limit intra venous treatment in this age’s category. All physicians should consider recurrence and prescribe prophylaxis [8–10]. The limits of our study were the absence of comparison between young people and aged people and the small number of cellulitis of the face and the upper limb. The cost of cellulitis was not calculated.

## Conclusion

To limit the cost of cellulitis and to decrease morbi-mortality in aged patients having cellulitis, health care officials must encourage aged persons to take care of their skin, to treat soft tissue infections and to prevent recurrence. Medical stuff must limit intra venous treatment and promote home care.

### What is known about this topic

- The frequency of cellulitis in elderly population;
- The predisposing factors in this age range;
- The recurrence of cellulitis.

### What this study adds

- Particularities of skin infection in elderly population;
- Characteristics of severe cellulitis;
- Promotion of Home Care.

## Competing interests

The authors declare no competing interest.
